# Acupuncture on “Huantiao” (GB30) and "Yanglingquan" (GB34) acupoints promotes nerve regeneration in mice model of peripheral nerve injury

**DOI:** 10.1016/j.ibneur.2023.08.004

**Published:** 2023-08-19

**Authors:** Qingjie Ji, Fangzhen Shan, Baojuan Zhang, Yunfeng Chen, Xianzhang Yang, Feng Gao, Xiang Li

**Affiliations:** a School of Acupuncture-moxibustion and Tuina, School of Health and Rehabilitation, Nanjing University of Chinese Medicine, Nanjing, Jiangsu Province, China; b Department of Rehabilitation, Affiliated Hospital of Jining Medical University, Jining, Shandong Province, China; c Medical Research Centre, Affiliated Hospital of Jining Medical University, Jining, Shandong Province, China; d Department of Rehabilitation, Jining Hospital of Traditional Chinese Medicine, Jining, Shandong Province, China; e Department of Neurology, Affiliated Hospital of Jining Medical University, Jining, Shandong Province, China

**Keywords:** Peripheral nerve injury, Acupuncture, Nerve regeneration, Schwann cells

## Abstract

**Objective:**

To investigate the effects of acupuncture on promoting nerve regeneration in mice with sciatic nerve crushed injury, an animal model of peripheral nerve injury (PNI).

**Methods:**

Acupuncture was performed on the "Huantiao" (GB30) and "Yanglingquan" (GB34) acupoints in PNI mice model for 2 weeks. Gait analysis, toe spreading test, electrophysiological test, toluidine blue staining and immunostaining of myelin basic protein (MBP), neurofilament-200 (NF200), p75 neurotrophin receptor (p75^NTR^), and growth associated protein-43 (GAP43) were respectively performed to investigate the effects of acupuncture on crushed sciatic nerve.

**Results:**

Acupuncture stimulation of “Huantiao” (GB30) and "Yanglingquan" (GB34) acupoints promoted the recovery of motor function and electrophysiological function in PNI mice model, which was indicated by a better gait level, toe spreading level and CMAP value in acupuncture group. The number of myelinated nerve fibers and the fluorescence intensity of MBP, NF200, p75^NTR^ and GAP43 staining demonstrated that the acupuncture stimulation promoted the regeneration of injured nerves in PNI mice model.

**Conclusion:**

Acupuncture significantly promoted the functional and morphological recovery of crushed sciatic nerve via promoting the expression of p75^NTR^ in Schwann cells.

## Highlights

• Acupuncture promotes the functional recovery of the mice model of peripheral nerve injury.
• Acupuncture causes significant electrophysiological and morphological changes of crushed sciatic nerve.
• Acupuncture modulates Schwann cells via upregulating the expression of p75^NTR^.

## 1 Introduction

Peripheral nerve injury (PNI) is clinically common, usually not life-threatening but can lead to a significant loss of function including motor dysfunction, hypoesthesia and skeletal muscle atrophy in the affected limbs (Liu et al., 2015). Current strategies for PNI are limited. As the glial cells in the peripheral nervous system, Schwann cells play critical roles in the process of peripheral nerve regeneration (Jessen and Mirsky, 2016, Jessen et al., 2015). Upon peripheral nerve injury, Schwann cells undergo proliferation and dedifferentiation and guide peripheral axonal regrowth and myelination. Presently, acupuncture is used as an important approach in clinical treatment for peripheral nerve injury. Studies revealed that acupuncture intervention could reduce the myelin outfoldings and axonal disintegration after peripheral nerve injury, furthermore, it could affect the nerve regeneration microenvironment to protect neurons thereby promoting neurological recovery (Cha et al., 2012, Gao et al., 2013, Hu et al., 2018). However, the effects of acupuncture on the Schwann cells in the process of nerve regeneration after peripheral nerve injury remains unknown.

The present study aimed to investigate the roles of acupuncture on the remyelination of SCs and nerve regeneration using a mice model of sciatic nerve crush and provide more evidence for the application of acupuncture in the treatment of PNI.

## 2 Experimental procedures

### 2.1 Experimental animals

Twenty healthy, wild-type male C57BL/6 mice, aged 8 weeks and weighed 22–25 g, were obtained from Jinan Pengyue Laboratory Animal Breeding CO., Ltd. (SCXK-Lu-2019–0003). All mice were housed in a conventional, ventilated environment with the temperature of 22–24 °C and relatively humidity of 40–60 % under 12 h light/12 h dark cycles. The bedding was replaced every 3–4 days. The mice were allowed to acclimate with free access to water and food for 7 days. The mice were then divided into the control group (n = 10) and the acupuncture group (n = 10) using a random number table. All animal procedures were conducted in compliance with the criteria of animal ethics and approved by the Medical Sciences Research Ethics Committee, Affiliated Hospital of Jining Medical University (2021B015).

### 2.2 Animal model

PNI was modeled using the clamp method according to the previous literature. After inhalation anesthesia with 2 % isoflurane/98 % O_2_ (RWB Life Science, Shenzhen, China), 2 cm^2^ skin of the ischial tuberosity was prepared. The mice were fixed in a prone position on the operating table, and then a sterile surgical towel was spread. Disinfection was routinely performed using iodophors. An approximately 1.5 cm oblique incision was created in the right thigh, and the muscle was separated by blunt dissection with tweezers. The sciatic nerve trunk and peripheral tissue were separated by toothpicks, and the sciatic nerve 5 mm away from the distal ischial tuberosity was clamped using a microscopic toothless vascular clamp (the blade is 1.3 mm in width), with the main force going to the 1/3 distal vascular clamp and a duration of 30 s. The nerve presented as transparent after. In the Control group, the sciatic nerve was exposed by blunt dissection without additional crushing. The incision was eventually closed with 6.0 sutures. To ensure homogeneity, all procedures were completed by the same experimenter and assistant. After modeling, the mice were resuscitated from anesthesia on a heating pad and then housed in separate cages.

### 2.3 Interventions

**Fig. 1:**
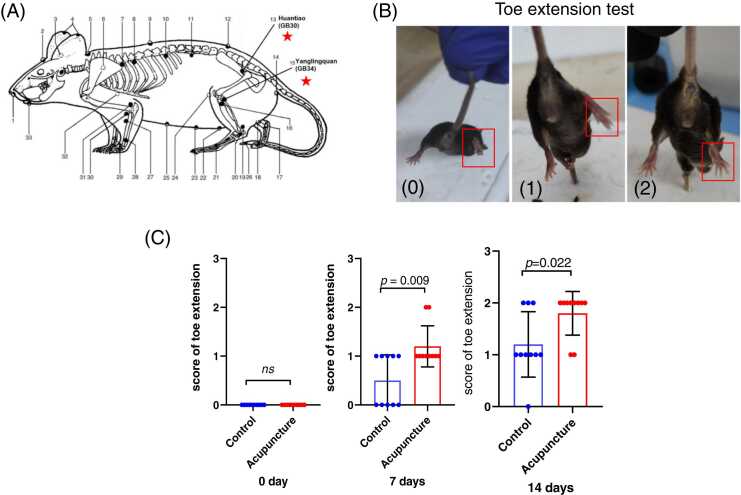
Acupuncture promoted the recovery of toe extension function in mice model of peripheral nerve injury. (A). Diagram illustrating the locations of acupuncture points in mice. The two acupoints used in the present study are marked with red stars. Equivalence of human acupoints in mice based on the Academic Department of China Association for Acupuncture and Moxibustion and the Jiangsu Institute of Traditional Chinese Medicine (Hua X et al., 1991). The figure was modified from Yanhu Xie (Xie et al., 2018). (B-C). Representative figure showing the quantitative points (0, 1 and 2, respectively) of toe extension test. The score of the acupuncture group (n = 10) was significantly higher than that of the control group (n = 10) at 7 and 14 days after intervention, but there was no difference at 0 day.

Three days post-modeling, mice of the acupuncture group were intervened by acupuncture using a disposable sterile acupuncture needle (13 mm × 0.18 mm, Wuxi Jiajian Medical Instrument, Jiangsu, China) after anesthesia with isoflurane. Traditional Chinese medicine believes in the concept of meridians and points as channels of energy flow in the body. According to this theory, if there is a problem in a particular area, selecting the corresponding acupuncture point along the meridian associated with that area can help in treatment. The "Huantiao" (GB30) acupoint and "Yanglingquan" (GB34) acupoint are points along the foot Shaoyang (Gallbladder) meridian, and they are commonly selected points for treating sciatic nerve-related issues in traditional Chinese medicine. The "Huantiao" (GB30) acupoint was localized to the upper rim of the posterior hip joint of the right hindlimb and the point of intersection between the greater trochanter of the femur and the bound portion of the coccyx and the ilium (external 1/3 and internal 2/3) of mice. The "Yanglingquan" (GB34) acupoint was localized to the depression below the anterior capitula fibula of the right hindlimb. The locations of acupuncture points in mice were shown in Fig. 1A. The "Huantiao" acupoint was directly punctured to a depth of 10 mm, resulting in the twitch of the lower extremity, while the "Yanglingquan" acupoint was punctured directly to a depth of 5 mm. The needles were left in place for 30 min. Acupuncture was performed once daily and six times per week for a total of 2 weeks.

### 2.4 Toe spreading test

After the mice were resuscitation for 1 h, a toe spreading test was performed. The toe spreading test was performed at 0, 7 and 14 days after acupuncture intervention. Refer to the test method of toe spreading recorded in the literature (Jessen and Mirsky, 2016)and make improvements. The specific methods are as follows: Hold the rat tail, let the mouse hold the cage with its forelimbs, wait for the mouse to be quiet, keep the limbs still for 5 s, and evaluate the right toe extension when the normal side toe is fully extended. The blind method was adopted in the experiment. Each evaluation was repeated 3 times, and the highest score was taken as the score of the toe extension. A 3-point score of was used. 0: no toe spread; 1 point: the toe has spread, but the spread is not complete; 2 points: All toes fully spread.

### 2.5 Gait analysis

Behavioral assessment was fulfilled by gait analysis based on the plantar imprinting using the Gait Analyzer (Zhongshi Science & Technology, Beijing, China) before and two days after modeling as well as two weeks after acupuncture intervention as previously described (Liu et al., 2023). By analyzing the gait of mice with plantar imprinted walking, the measured indexes include: stand (duration of the paw contacting runway), max contact area (area of the paw contacting runway during stance phase), and stance width (distance between footprints on both sides). The sciatic function index (SFI) was calculated to monitor recovery after sciatic nerve injury. The SFI was calculated according to the following formula:

SFI= −38.3((EPL - NPL)/NPL) + 109.5 ((ETS-NTS)/NTS) + 13.3((EITS - NITS)/NITS) - 8.8.

All these measurements were taken from the experimental (injured) and normal (uninjured) sides, as following, EPL (experimental print length), NPL (normal print length), ETS (experimental toe spread), NTS (normal toe spread), EITS (experimental intermediary toe spread) and NITS (normal intermediary toe spread). The lower SFI indicates more severe functional impairment of sciatic nerve.

### 2.6 Electrophysiological test

Sciatic nerve conduction was tested two weeks after intervention. At first, the sciatic nerve was fully exposed after anesthesia with isoflurane. The recording electrode was placed in the gastrocnemius muscle belly, and the reference electrode was placed in the toe, with the grounding wire connected to the tails (the skin with surface electrode was prepared for further treatment). After the stimulation needle electrode (Repusi, Nr.10.1–37, Suzhou, China) was well-connected to the nerve trunk, the sciatic nerve was stimulated by a single pulse until maximum amplitude of CMAP, followed by 20 % increase in pulse intensity to ensure maximum amplitude (duration: 0.1 ms, repetition rate: 0.5 Hz). The latency period was measured from the occurrence of stimulation artifacts to the action potential initiation, if the nerve trunk action potential amplitudes were measured from the highest point to the lowest point of the waveform. The whole process was completed at routine temperature within 30 min

### 2.7 Morphological examination

Toluidine blue staining was performed to observe the morphological changes of axon and myelin sheath in nerve fibers. After anesthesia with isoflurane, the thoracic cavity was cut to expose the heart. The ascending aorta was punctured via the apex using an infusion needle, while the right atrial appendage was instantly cut. 20 ml PBS was infused, followed by 20 ml instant fixative solution containing 4 % paraformaldehyde and 2.5 % glutaraldehyde. The femoral skin was cut to expose the sciatic nerve, from which a 2 mm segment was sampled and fixed in the fixative solution for 24 h. The segment underwent a second fixation in 1 % osmium tetroxide and was then dehydrated, paraffin-embedded, sectioned, xylene-dewaxed, washed with different concentrations of alcohol, stained with toluidine blue dye solution, xylene-transparentized, and sealed. The structure and permutation distribution of axon and myelin sheath in nerve fibers were observed under an optical microscope (Zeiss, Germany), and the number of myelinated nerve fibers was calculated using the Image J software.

### 2.8 Fluorescent immunohistochemistry

After perfusion using 4 % paraformaldehyde, the dissected sciatic nerve was further fixed in 4 % paraformaldehyde for 2 h, washed with PBS, dehydrated in 30 % sucrose solution for 24 h, embedded in OCT and then sectioned to 6 µm sections. The sections were blocked with 10 % goat serum (Solarbio, Beijing, China) and 0.3 % Triton X-100 in PBS at room temperature for 1 h, followed by overnight incubation with primary antibodies at 4 °C, including anti-myelin basic protein (MBP) (1:200), anti-neurofilament-200 (NF200) (1:1000), anti-p75 neurotrophin receptor (p75^NTR^) (1:200), and anti-growth-associated protein-43 (GAP43) (1:200) (Thermo Fisher Scientific, USA). After three washing using PBS, secondary antibodies were added for additional incubation at room temperature for 2 h. The sections were then sealed with anti-fluorescence quenching sealing tablets and observed under a Fluorescence microscope (Zeiss, Germany). Images were captured, and the fluorescence intensity of the target protein was measured using the Image J software.

### 2.9 Statistical analysis

Graphpad Prism 6.0 software was applied for statistical analysis. Data that conformed to normal distribution including SFI, electrophysiological indices, density of myelinated nerve fibers, fluorescence intensity of NF200, MBP, p75NTR and GAP43 were analyzed by unpaired t-test. Other data that did not conform to normal distribution presented as median (interquartile range) and analyzed with the Mann-Whitney *U* test. A two-sided p value of <0.05 were considered as statistically significant.

## 3 Results

### 3.1 Acupuncture improved the motor function of sciatic nerve of the mice after crush injury

As shown in Fig. 1B and C, the score of the toe extension in all mice was zero at 0 day, indicating sciatic nerve was injured**. T**here were significantly more quantitative points in the acupuncture group (n = 10) than the control group (n = 10) at both 7 days and 14 days after acupuncture treatment.

**Fig. 2:**
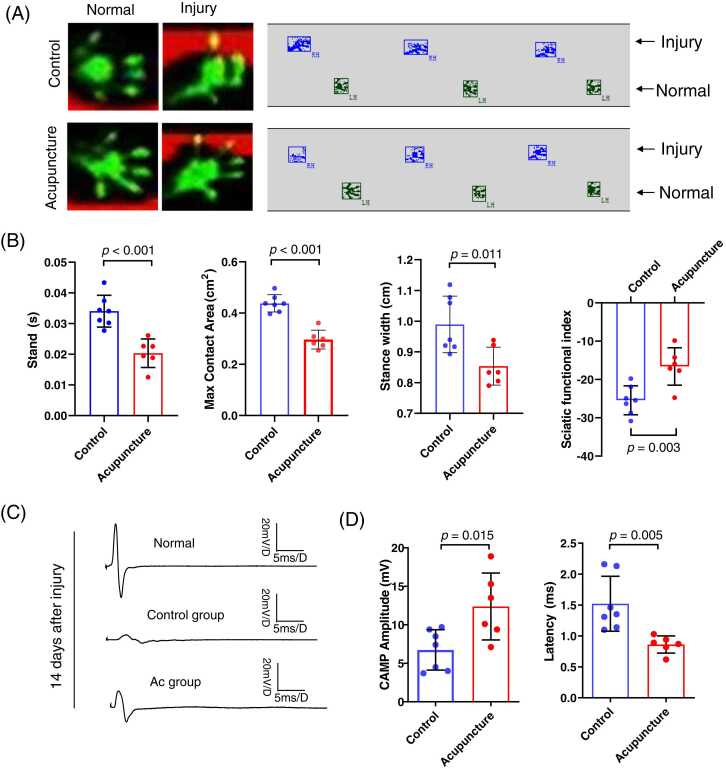
Acupuncture promoted the recovery of motor function and electrophysiological conduction in mice model of peripheral nerve injury. (A). Representative images of footprints of mice from acupuncture group (n = 10) and control group (n = 10). (B). The gait analysis showed that the stand time (s), max contact area (cm²) and stance width (cm) were significantly higher in acupuncture group than the control group, and the SFI score of the acupuncture group was significantly lower than that of the control group. (C-D). Electrophysiological test showed that the amplitude of CAMP was significantly higher in acupuncture group than that in the control group, and the prolonged latency of CAMP was significantly lower in acupuncture group than that in the control group.

As shown in Fig. 2, gait analysis showed the motor function of mice model of PNI in acupuncture group was significantly improved than the control group, including the stand time (0.03405 ± 0.0137 s vs 0.02035 ± 0.002751 s, *p*<0.001), max contact area (0.4380 ± 0.1414 cm^2^ vs 0.2966 ± 0.1414 cm^2^, *p*<0.001), stance width (0.989 ± 0.1356 cm vs 0.8537 ± 0.04439 cm, *p*=0.011), SFI (–16.59 ± 2.400 vs –25.43 ± 8.840, *p*=0.0036) (Fig. 2A and B).

In comparison to the control group, the amplitude of CMAP of sciatic nerve of the mice model was remarkably increased in mice after acupuncture (12.38 ± 4.342 mV vs 6.743 ± 2.618 mV, *p*=0.015), while the latency period was significantly shortened (0.8617 ± 0.1382 ms vs 1.521 ± 0.4433 ms, *p*=0.005) (Fig. 2C and D).

### 3.2 Morphology changes in crushed sciatic nerve after acupuncture

**Fig. 3:**
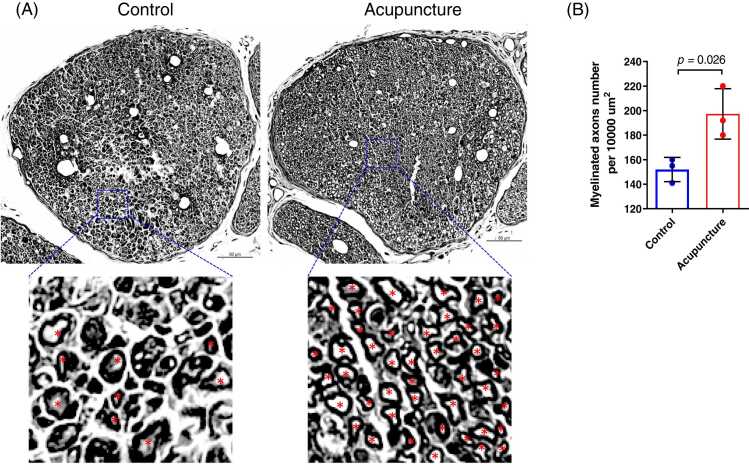
Toluidine blue staining of the crushed sciatic nerve. (A). The toluidine blue staining of sciatic nerves from acupuncture and control group mice at 14 days of acupuncture were shown respectively. Red asterisk used to label fibers with intact myelin sheath. Bar = 50 µm. (B). The number of myelinated fibres in sciatic nerves was significantly higher in acupuncture group than that in the control group.

**Fig. 4:**
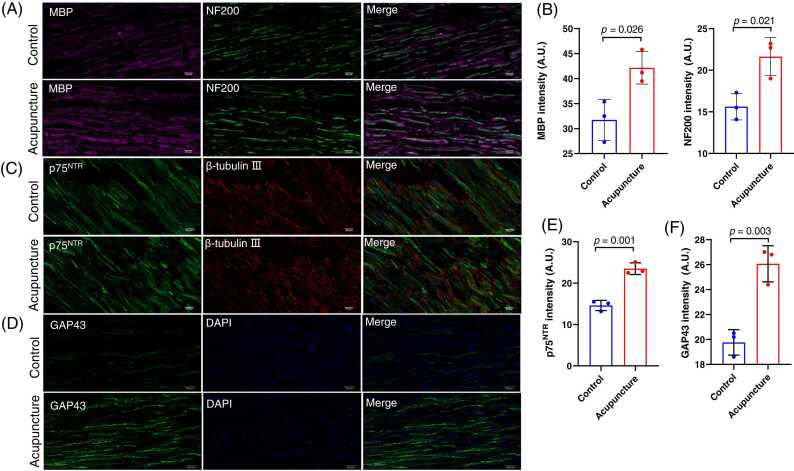
Immunostaining of MBP, NF200, p75^NTR^ and GAP43 in crushed sciatic nerves. (A-F). Immunostaining of MBP, NF200, p75^NTR^ and GAP43 were performed at 14 days of acupuncture treatment to investigate the therapeutic effects of acupuncture on crushed the sciatic nerves of mice in acupuncture (n = 3) and control group (n = 3). The fluorescence intensity of MBP, NF200, p75^NTR^, and GAP43 were significant higher in acupuncture group than that in the control group. Bar = 20 µm.

Toluidine blue staining was performed to observe morphological changes of sciatic nerves. In the Control group, the sciatic nerve fibers and myelin sheathes were disarranged, and disintegration of the myelin sheath and axon was observed. While in the Acupuncture group, the sciatic nerve fibers tended to be arranged regularly with only few myelin sheathes dislodged (Fig. 3A). In addition, the number of myelinated nerve fibers was significantly higher in the Acupuncture group than that in the Control group (*p*=0.0261) (Fig. 3B). MBP, GAP43, NF200, and p75^NTR^ are important markers of PNI repair (Gonçalves et al., 2019; Liu et al., 2015; Gaetani et al., 2019). As shown in Fig. 4, there were significantly more fluorescence intensity of MBP, NF200, p75^NTR^ and GAP43 staining in crushed sciatic nerves of mice in acupuncture group than control group.

## 4 Discussion

Early PNI presents mainly with pain, numbness and dyskinesia, consistent with the category of "impediment pattern" of traditional Chinese medicine (TCM); while in the later phase, the loss of neurotrophic effects lead to muscle wasting and weakness, which belongs to the "wilting pattern" of TCM. In the present study, we selected the "Huantiao" and "Yanglingquan" acupoints for acupuncture intervention. The "Huantiao" acupoint is localized to the foot lesser yang gallbladder meridian, the intersection of foot lesser yang meridian and foot greater yang meridian. The place that meridians pass through demands priority in your diagnosis. PNI mostly presents with atrophy and weakness of the lower extremity, where the foot lesser yang meridian and foot greater yang meridian pass through. The "Yanglingquan" acupoint is localized to the meeting place of the sinew (one of the eight meeting points) as the acupoint of foot lesser yang gallbladder meridian. PNI is regarded as a result of injury of meridians, and thus the "Huantiao" and "Yanglingquan" acupoints were selected for acupuncture intervention in this study. The two acupoints used in the present study are marked with red stars. Equivalence of human acupoints in mice based on the Academic Department of China Association for Acupuncture and Moxibustion and the Jiangsu Institute of Traditional Chinese Medicine (Hua et al., 1991). The figure was modified from Xie et al. (2018). Acupuncture significantly promoted the peripheral nerve repairment after injury (Zhang et al., 2021). Previous studies have proved that acupuncture was conducive to myelin reconstruction and axonal regrowth by enhancing expression of multiple neurotrophic factors, and regulating axonal growth-directed molecules (Fei et al., 2019; Liu et al., 2015; Song et al., 2020; Yang et al., 2019). However, the effect of acupuncture on Schwann cells after peripheral nerve injury has scarcely been covered yet.

We found that acupuncture significantly increased the amplitude of CMAP and shortened the latency of the crushed sciatic nerve. Being consistently, there was significantly higher levels of MBP and NF200 immunostaining in the crushed sciatic nerve of mice with acupuncture treatment. The electrophysiological test and the immunostaining study both demonstrated that acupuncture significantly promoted the nerve regeneration of the crushed sciatic nerve. As a member of the tumor necrosis factor receptor superfamily, P75^NTR^ is a transmembrane protein expressed in various tissues, including the nervous system (Cragnolini et al., 2009, Meeker and Williams, 2015). P75^NTR^ acts as a co-receptor for several neurotrophins, including nerve growth factor, brain derived neurotrophic factor, and neurotrophin-3, a group of proteins that regulate the development, survival and plasticity of neurons (Chao, 2003). In normal peripheral nervous system, p75^NTR^ is expressed at low levels in Schwann cells; however, after peripheral nerve injury, the expression of p75 ^NTR^ dramatically increases to play a role in promoting nerve regeneration by aiding in the clearance of myelin debris and the attraction of Schwann cells to the injury site. In addition, p75 ^NTR^ can promote axonal growth and remyelination by activating intracellular signaling pathways that enhance Schwann cell proliferation and differentiation. In this study, we overserved significant increase of p75 ^NTR^ in the crushed sciatic nerve of mice with acupuncture treatment, which indicates that acupuncture may promote the regeneration of crushed sciatic nerve via modulating the Schwann cells. Being expressed predominantly in neuron axons, GAP-43 is a neuronal phosphoprotein that plays an important role in axonal growth and is upregulated during nerve regeneration (Liu et al., 2010). It has been shown that increasing the expression of GAP-43 can enhance axonal regeneration and functional recovery in various nerve injury models, including peripheral nerve injury. In addition to the increase of p75 ^NTR^ in the crushed sciatic nerve, we also found that acupuncture treatment significantly promoted the expression of GAP43. Notably, it remains unknown whether the upregulation of GAP43 in crushed sciatic nerve is directly caused by acupuncture stimulus or indirectly induced by p75 ^NTR^-related remyelination, which deserves further study.

To conclude, acupuncture significantly promoted the functional and morphological recovery of crushed the sciatic nerve by modulating the Schwann cells. Our study provides new evidences for clinical application of acupuncture in the treatment of peripheral nerve. Because the effects of acupuncture on promoting the peripheral nerve regeneration are usually slow, further study is necessary to elucidate the definite mechanism of acupuncture on Schwann cells for developing strategies for enhancing the efficacy.

## Funding

This work was funded by the Key Science and Technology Support Program of Jining, China (Nos. 2022YXNS168 and 2020JKNS011) and the Research Fund for Lin He’s Academician Workstation of New Medicine and Clinical Translation in Jining Medical University, China (No. JYHL2021FMS20).

## CRediT authorship contribution statement

Study concept and design: **Xiang Li**. Data acquisition: **Qingjie Ji**, **Fangzhen Shan**, **Baojuan Zhang**, **Yunfeng Chen**, **Xianzhang Yang** and **Feng Gao**. Data interpretation: all authors. Statistical analysis: **Qingjie Ji**. Drafting the manuscript: **Qingjie Ji**. Critical reading and revision of the manuscript: **Xiang Li**.

## Conflict of interest

The authors declare no conflict of interest.

## Data Availability

The original contributions presented in this study are included in the article, further inquiries can be directed to the corresponding authors.
